# 8-Chloro-2-methyl­quinoline

**DOI:** 10.1107/S1600536809020194

**Published:** 2009-06-06

**Authors:** Tian-Quan Wu, Jian-Hua Wang, Fang Shen, Ai-Xi Hu

**Affiliations:** a College of Chemistry and Chemical Engineering, Hunan University, 410082 Changsha, People’s Republic of China

## Abstract

In the title compound, C_10_H_8_ClN, the crystal packing shows π–π stacking between the heterocyclic ring and the aromatic ring, with a centroid–centroid distance of 3.819 Å. The crystal studied was a racemic twin, the ratio of the twin components being 0.65 (7):0.35 (7).

## Related literature

The title compound is an important inter­mediate in the pharmaceutical industry, see: Shen & Hartwig (2006 ▶); Ranu *et al.* (2000 ▶); Lee & Hartwig (2005 ▶).
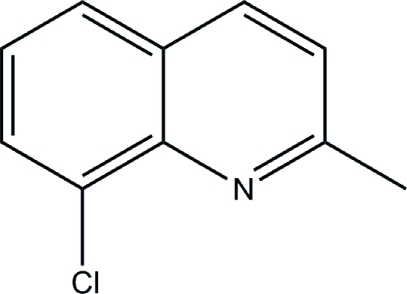


## Experimental

### 

#### Crystal data

C_10_H_8_ClN
*M* *_r_* = 177.62
Orthorhombic, 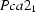

*a* = 12.7961 (9) Å
*b* = 5.0660 (4) Å
*c* = 13.1181 (9) Å
*V* = 850.38 (11) Å^3^
*Z* = 4
Mo *K*α radiation
μ = 0.39 mm^−1^
*T* = 173 K
0.47 × 0.46 × 0.23 mm

#### Data collection

Bruker SMART 1000 CCD diffractometer
Absorption correction: multi-scan (*SADABS*; Sheldrick, 2004 ▶) *T*_min_ = 0.840, *T*_max_ = 0.917
3943 measured reflections
1821 independent reflections
1703 reflections with *I* > 2σ(*I*)
*R*_int_ = 0.016

#### Refinement

*R*[*F* ^2^ > 2σ(*F* ^2^)] = 0.029
*wR*(*F* ^2^) = 0.075
*S* = 1.09
1821 reflections
111 parameters
1 restraint
H-atom parameters constrained
Δρ_max_ = 0.20 e Å^−3^
Δρ_min_ = −0.16 e Å^−3^

### 

Data collection: *SMART* (Bruker, 2001 ▶); cell refinement: *SAINT-Plus* (Bruker, 2003 ▶); data reduction: *SAINT-Plus*; program(s) used to solve structure: *SHELXS97* (Sheldrick, 2008 ▶); program(s) used to refine structure: *SHELXL97* (Sheldrick, 2008 ▶); molecular graphics: *SHELXTL* (Sheldrick, 2008 ▶); software used to prepare material for publication: *SHELXL97*.

## Supplementary Material

Crystal structure: contains datablocks I, global. DOI: 10.1107/S1600536809020194/bt2969sup1.cif

Structure factors: contains datablocks I. DOI: 10.1107/S1600536809020194/bt2969Isup2.hkl

Additional supplementary materials:  crystallographic information; 3D view; checkCIF report

## Figures and Tables

**Fig. 1.**
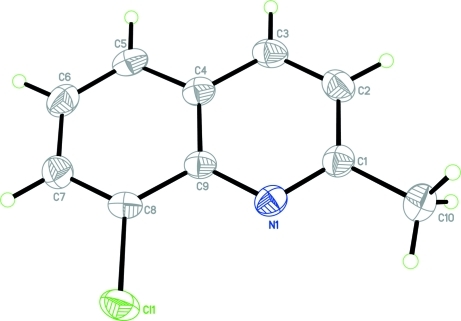
Molecular structure of the title compound showing 50% probability displacement ellipsoids.

## supplementary crystallographic information

### Comment

The structure of the title compound, 8-chloro-2-methylquinoline, is shown in Fig 1. It is an important intermediate of medecine industry (Shen *et al.*, 2006; Ranu *et al.*, 2000; Lee *et al.*, 2005). The crystal  packing shows π-π stacking between the N containing aromatic ring and the aromatic ring with the chloro substituent with a centroid-centroid distance of 3.819Å.

### Experimental

A solution of 13 g of 2-chloroaniline in 200 mL chlorobenzene and 0.5 g of *p*-toluenesulfonic acid was heated to 393 K.   14 g of crotonaldehyde were added dropwise with in 1 h, then refluxed for 2 h. The solution was concentrated under reduced pressure to give rude product, which was then recrystallizated from dimethylbenzene to get 10 g of the product as a white solid. The yield was 57%. Crystals suitable for X-ray structure determination were obtained by slow evaporation of an ethanol solution at room temperature.

### Refinement

H atom were positioned geometrically (C_aromatic_—H = 0.95 Å, C_methyl_—H = 0.98 Å)   and refined as riding with U_iso_(H) = 1.2U_eq_(C_aromatic_) or U_iso_(H) = 1.5U_eq_(C_methyl_). The crystal under investigation turned out to be a racemic twin with a ratio of the twin components of 0.65 (7) to 0.35 (7).

### Crystal data

**Table:**

C_10_H_8_ClN	*D*_x_ = 1.387 Mg m^−^^3^
*M**_r_* = 177.62	Melting point: 333 K
Orthorhombic, *P**c**a*2_1_	Mo *K*α radiation, λ = 0.71073 Å
Hall symbol: P 2c -2ac	Cell parameters from 2761 reflections
*a* = 12.7961 (9) Å	θ = 3.1–27.0°
*b* = 5.0660 (4) Å	µ = 0.39 mm^−^^1^
*c* = 13.1181 (9) Å	*T* = 173 K
*V* = 850.38 (11) Å^3^	Block, colourless
*Z* = 4	0.47 × 0.46 × 0.23 mm
*F*(000) = 368	

### Data collection

**Table:**

Bruker SMART 1000 CCD diffractometer	1821 independent reflections
Radiation source: fine-focus sealed tube	1703 reflections with *I* > 2σ(*I*)
graphite	*R*_int_ = 0.016
ω scans	θ_max_ = 27.1°, θ_min_ = 3.1°
Absorption correction: multi-scan (*SADABS*; Sheldrick, 2004)	*h* = −16→16
*T*_min_ = 0.840, *T*_max_ = 0.917	*k* = −2→6
3943 measured reflections	*l* = −15→16

### Refinement

**Table:**

Refinement on *F*^2^	Primary atom site location: structure-invariant direct methods
Least-squares matrix: full	Secondary atom site location: difference Fourier map
*R*[*F*^2^ > 2σ(*F*^2^)] = 0.029	Hydrogen site location: inferred from neighbouring sites
*wR*(*F*^2^) = 0.075	H-atom parameters constrained
*S* = 1.09	*w* = 1/[σ^2^(*F*_o_^2^) + (0.0403*P*)^2^ + 0.17*P*] where *P* = (*F*_o_^2^ + 2*F*_c_^2^)/3
1821 reflections	(Δ/σ)_max_ = 0.004
111 parameters	Δρ_max_ = 0.20 e Å^−^^3^
1 restraint	Δρ_min_ = −0.16 e Å^−^^3^

### Special details

**Table:**

Experimental. MS (m/*z*):*M*+ 177. ^1^H NMR(CDCl_3_,400 MHz,delta dppm): 2.83(s,3*H*,CH_3_), 7.38(m,2*H*,quinoline 3,6-H), 7.80(d, J=7.2 Hz,1*H*, quinoline 7-H),8.03(d, J =8.0 Hz,1*H*,quinoline 5-H), 8.00(d,J = 8.4 Hz, 1H,quinoline 4-H)
Geometry. All e.s.d.'s (except the e.s.d. in the dihedral angle between two l.s. planes) are estimated using the full covariance matrix. The cell e.s.d.'s are taken into account individually in the estimation of e.s.d.'s in distances, angles and torsion angles; correlations between e.s.d.'s in cell parameters are only used when they are defined by crystal symmetry. An approximate (isotropic) treatment of cell e.s.d.'s is used for estimating e.s.d.'s involving l.s. planes.
Refinement. Refinement of *F*^2^ against ALL reflections. The weighted *R*-factor *wR* and goodness of fit *S* are based on *F*^2^, conventional *R*-factors *R* are based on *F*, with *F* set to zero for negative *F*^2^. The threshold expression of *F*^2^ > σ(*F*^2^) is used only for calculating *R*-factors(gt) *etc*. and is not relevant to the choice of reflections for refinement. *R*-factors based on *F*^2^ are statistically about twice as large as those based on *F*, and *R*- factors based on ALL data will be even larger.

### Fractional atomic coordinates and isotropic or equivalent isotropic displacement parameters (Å^2^)

**Table:**

	*x*	*y*	*z*	*U*_iso_*/*U*_eq_	
Cl1	0.13075 (3)	−0.06692 (9)	−0.13065 (4)	0.03616 (14)	
C1	0.33097 (14)	0.5106 (4)	0.00528 (15)	0.0286 (4)	
C2	0.33961 (16)	0.5667 (4)	0.11114 (16)	0.0336 (4)	
H2	0.3873	0.6971	0.1345	0.040*	
C3	0.27923 (15)	0.4323 (3)	0.17875 (15)	0.0323 (4)	
H3	0.2846	0.4675	0.2497	0.039*	
C4	0.20807 (14)	0.2386 (3)	0.14243 (14)	0.0277 (4)	
C5	0.14115 (15)	0.0969 (4)	0.20801 (15)	0.0327 (4)	
H5	0.1427	0.1293	0.2793	0.039*	
C6	0.07432 (15)	−0.0864 (4)	0.16908 (16)	0.0349 (4)	
H6	0.0285	−0.1792	0.2134	0.042*	
C7	0.07260 (15)	−0.1394 (4)	0.06385 (16)	0.0330 (4)	
H7	0.0266	−0.2701	0.0377	0.040*	
C8	0.13698 (14)	−0.0033 (4)	−0.00123 (15)	0.0271 (4)	
C9	0.20665 (13)	0.1930 (3)	0.03548 (13)	0.0248 (3)	
C10	0.39634 (17)	0.6615 (5)	−0.06992 (17)	0.0393 (5)	
H10A	0.4700	0.6137	−0.0613	0.059*	
H10B	0.3877	0.8513	−0.0582	0.059*	
H10C	0.3740	0.6181	−0.1394	0.059*	
N1	0.26803 (12)	0.3289 (3)	−0.03180 (11)	0.0268 (3)	

### Atomic displacement parameters (Å^2^)

**Table:**

	*U*^11^	*U*^22^	*U*^33^	*U*^12^	*U*^13^	*U*^23^
Cl1	0.0393 (2)	0.0441 (3)	0.0251 (2)	−0.00280 (19)	−0.0036 (2)	−0.0074 (2)
C1	0.0279 (9)	0.0261 (8)	0.0319 (10)	0.0032 (7)	−0.0008 (8)	0.0037 (7)
C2	0.0343 (10)	0.0291 (10)	0.0374 (11)	−0.0015 (8)	−0.0081 (8)	−0.0030 (8)
C3	0.0397 (10)	0.0305 (9)	0.0266 (9)	0.0039 (8)	−0.0052 (8)	−0.0054 (7)
C4	0.0320 (9)	0.0253 (9)	0.0259 (9)	0.0060 (7)	−0.0009 (7)	−0.0001 (7)
C5	0.0412 (10)	0.0344 (10)	0.0223 (9)	0.0063 (8)	0.0002 (8)	0.0031 (7)
C6	0.0339 (10)	0.0382 (11)	0.0325 (10)	−0.0010 (8)	0.0045 (8)	0.0086 (8)
C7	0.0297 (9)	0.0334 (10)	0.0360 (10)	−0.0033 (8)	−0.0023 (8)	0.0029 (8)
C8	0.0294 (9)	0.0310 (8)	0.0209 (9)	0.0033 (7)	−0.0029 (7)	−0.0013 (7)
C9	0.0253 (8)	0.0245 (8)	0.0247 (9)	0.0056 (7)	−0.0025 (7)	0.0005 (6)
C10	0.0408 (10)	0.0375 (10)	0.0396 (12)	−0.0067 (10)	0.0004 (9)	0.0082 (9)
N1	0.0265 (7)	0.0274 (7)	0.0264 (8)	0.0039 (6)	0.0013 (6)	0.0030 (6)

### Geometric parameters (Å, °)

**Table:**

Cl1—C8	1.730 (2)	C5—H5	0.9500
C1—N1	1.316 (3)	C6—C7	1.407 (3)
C1—C2	1.422 (3)	C6—H6	0.9500
C1—C10	1.503 (3)	C7—C8	1.372 (3)
C2—C3	1.359 (3)	C7—H7	0.9500
C2—H2	0.9500	C8—C9	1.420 (3)
C3—C4	1.421 (2)	C9—N1	1.367 (2)
C3—H3	0.9500	C10—H10A	0.9800
C4—C5	1.410 (3)	C10—H10B	0.9800
C4—C9	1.422 (2)	C10—H10C	0.9800
C5—C6	1.362 (3)		
			
N1—C1—C2	123.25 (18)	C7—C6—H6	119.7
N1—C1—C10	116.99 (18)	C8—C7—C6	120.36 (18)
C2—C1—C10	119.76 (18)	C8—C7—H7	119.8
C3—C2—C1	119.55 (18)	C6—C7—H7	119.8
C3—C2—H2	120.2	C7—C8—C9	121.19 (18)
C1—C2—H2	120.2	C7—C8—Cl1	119.30 (15)
C2—C3—C4	119.44 (18)	C9—C8—Cl1	119.49 (15)
C2—C3—H3	120.3	N1—C9—C8	119.62 (16)
C4—C3—H3	120.3	N1—C9—C4	123.21 (16)
C5—C4—C3	122.42 (17)	C8—C9—C4	117.16 (16)
C5—C4—C9	120.76 (17)	C1—C10—H10A	109.5
C3—C4—C9	116.82 (16)	C1—C10—H10B	109.5
C6—C5—C4	119.98 (18)	H10A—C10—H10B	109.5
C6—C5—H5	120.0	C1—C10—H10C	109.5
C4—C5—H5	120.0	H10A—C10—H10C	109.5
C5—C6—C7	120.54 (18)	H10B—C10—H10C	109.5
C5—C6—H6	119.7	C1—N1—C9	117.70 (16)
			
N1—C1—C2—C3	−1.4 (3)	Cl1—C8—C9—N1	−0.1 (2)
C10—C1—C2—C3	179.05 (18)	C7—C8—C9—C4	1.1 (2)
C1—C2—C3—C4	−0.3 (3)	Cl1—C8—C9—C4	179.55 (13)
C2—C3—C4—C5	−178.29 (18)	C5—C4—C9—N1	178.44 (15)
C2—C3—C4—C9	1.5 (2)	C3—C4—C9—N1	−1.4 (2)
C3—C4—C5—C6	179.90 (17)	C5—C4—C9—C8	−1.2 (2)
C9—C4—C5—C6	0.1 (3)	C3—C4—C9—C8	178.99 (15)
C4—C5—C6—C7	1.1 (3)	C2—C1—N1—C9	1.5 (3)
C5—C6—C7—C8	−1.2 (3)	C10—C1—N1—C9	−178.87 (16)
C6—C7—C8—C9	0.1 (3)	C8—C9—N1—C1	179.50 (16)
C6—C7—C8—Cl1	−178.36 (15)	C4—C9—N1—C1	−0.1 (2)
C7—C8—C9—N1	−178.55 (17)		
